# Ecological competition in the oral mycobiome of Hispanic adults living in Puerto Rico associates with periodontitis

**DOI:** 10.1080/20002297.2024.2316485

**Published:** 2024-02-21

**Authors:** Kimil Acosta-Pagán, Benjamín Bolaños-Rosero, Cynthia Pérez, Ana P. Ortíz, Filipa Godoy-Vitorino

**Affiliations:** a Department of Microbiology and Medical Zoology, Medical Sciences Campus, University of Puerto Rico, San Juan, Puerto Rico; b Graduate School of Public Health, Medical Sciences Campus, University of Puerto Rico, San Juan, Puerto Rico; c Division of Cancer Control and Population Sciences, University of Puerto Rico Comprehensive Cancer Center, San Juan, Puerto Rico, USA

**Keywords:** Mycobiota, oral cavity, Hispanics, periodontitis, Candida

## Abstract

**Background:** Fungi are a major component of the human microbiome that only recently received attention. The imbalance of indigenous fungal communities and environmental fungi present in the oral cavity may have a role in oral dysbiosis, which could exacerbate oral inflammatory diseases.

**Methods:** We performed a cross-sectional study and recruited 88 participants aged 21 to 49 from sexually transmitted infection clinics in Puerto Rico. A full-mouth periodontal examination following the NHANES protocol defined periodontal severity (CDC/AAP). ITS2 (fungal) genes were amplified and sequenced for mycobiota characterization of yeast and environmental fungi. Environmental outdoor spore levels were measured daily by the American Academy of Allergy Asthma and Immunology San Juan station and defined by quartiles as spore scores.

**Results:** Our data indicate polymicrobial colonization of yeast and environmental fungi in the oral cavity. Dominant taxa associated with periodontal disease included *Saccharomyces cerevisiae*, *Rigidoporus vinctus*, and *Aspergillus penicilloides*, while *Candida albicans* were found to be ubiquitous. Fungal aerosols were found to impact the oral cavity biofilm, likely due to competition and neutralization by inhaled outdoor and indoor fungal spores.

**Conclusion:** To our knowledge, this is the first report showcasing the ecological competition of measured outdoor environmental fungi with the human oral mycobiota.

## Introduction

Bacterial and fungal communities play an essential role in developing the oral biofilm. These communities participate in infectious dysbiotic processes of many diseases, such as dental caries, gingivitis, and periodontitis, which could lead to oral cancer. Gingivitis and periodontitis are common bacterial infections caused by host immune responses against pathogenic microorganisms, leading to inflammation and dysbiosis [1]. While gingivitis is a mild reversible inflammation, if left untreated, it can develop into periodontitis, an irreversible disease that causes chronic inflammation [2]. This chronic inflammation produces inflammatory mediators that can induce mutagenesis, cell proliferation, and cell degeneration. This process is one of the major causes of pathogens taking advantage of several oral surfaces, provoking teeth loss, destruction of supporting structures, and gingival recession [3]. Several factors have been associated with the exacerbation of periodontal disease, for instance, smoking, heavy alcohol consumption, diet, sexual practices, and HPV infection [4].

Microorganisms cause about 20% of human cancers [5]. While microbes in saliva are not considered a direct agent of oral disease, ecological changes in gingival crevice physiological conditions may affect microbiota composition. Research has focused mainly on bacterial communities; however, fungal communities remain understudied compared to the bacterial biota [6]. For instance, fungi have been implicated in exacerbating several human diseases, but their potential role as modulators remains unexplored. It has been demonstrated that filamentous fungi and yeasts can form biofilms in both biotic and abiotic surfaces, only exacerbating periodontal disease and other chronic inflammation conditions by making them more resistant to treatments [7].

Many *Candida* species are considered commensal in the oral cavity [8]. However, individuals with pre-existing health conditions, such as diabetes, HIV, periodontal disease, and cancer, are at a higher risk of developing oral candidiasis [7]. This infection, also known as oral thrush, often occurs when *Candida* overgrows in individuals with a weakened immune system, allowing the formation of biofilms by adhering to the epithelial cells in the oral cavity [7]. Although *Candida albicans* has been highly associated with oral thrush, many species like *C. glabrata, C. krusei, C. parapsilosis, C. pseudotropicalis*, and *C. tropicalis* have also been linked to candidiasis [9]. Formation of these biofilms is of utmost importance, as they can grow inside periodontal pockets. This process aids inflammatory responses and permits other pathogenic and opportunistic microbes to infiltrate tissues, creating more bone loss [10]. Studying polymicrobial associations is of clinical concern regarding the synergies of bacterial and fungal biofilms, with infections expected to be more severe and burdensome to treat with antimicrobials. Hence, knowing the relationship between pathogenic bacteria and fungi implies the possible discovery of treatment strategies for periodontal disease and preventing oral complications.

To our knowledge, no other study before has simultaneously associated the levels of environmental outdoor fungal spores matched daily with oral fungi measured in the oral cavity. We investigated the composition of the oral fungal community diversity and its relationship with periodontal disease severity and periodontal risk factors among Hispanic adults. Additionally, we matched environmental spore levels and evaluated how oral mycological diversity is associated with spore counts from the recruitment days. Our study establishes possible ecological implications of outdoor fungal spores’ aspiration on the resident mycobiota of the oral cavity.

## Materials and methods

### Description of the study population

This cross-sectional study recruited sexually active Hispanic adults living in Puerto Rico, given the high prevalence of high-risk sexual practices in the adult population in Puerto Rico [11]. Participants with high-risk sexual practices and coming to STI clinics may have a higher burden of oral diseases such as periodontitis [12]. We hypothesized that changes in oral fungi in participants with STI-related concerns would be associated with periodontal severity status. Eligible participants were aged 21–49 years old, and mentally capable of participating in the study. Exclusion criteria included those characteristics that could impact the microbiome, such as HIV-positive status, pregnant women, hormonal contraceptive use, use of antibiotics in the last 2 months, postmenopausal status, depression or post-traumatic stress disorder diagnostic, and pre-existing heart conditions such as endocarditis, prosthetic cardiac valves, cardiac transplant, valvular heart disease, or congenital heart defect.

### Recruitment and data collection procedures

This study was approved by the Institutional Review Board of the University of Puerto Rico Comprehensive Cancer Center (protocol 2018-01-01). All subjects provided written informed consent. Participants were recruited from two sexually transmitted infection (STI) clinics and recruited in Alliance in San Juan, Puerto Rico, promotion on social media and person-to-person promotion. Study participants completed procedures at the Hispanic Alliance for Clinical and Translational Research (ALLIANCE; U54GM133807).

Sociodemographic, lifestyle, and clinical information were collected through an interviewer-administered questionnaire, and measurements included sex, age, oral hygiene, smoking, alcohol consumption, marihuana usage, and oral sex practices. This questionnaire also included medical and dental history, comorbidities, and diet. Information regarding drug use and sexual behaviors was assessed using an audio computer-assisted self-interview (ACASI) [13]. Clinical procedures included saliva collection for mycobiome characterization, dental evaluation for periodontal disease assessment, and anthropometric measurements to determine body mass index (BMI, kg/m2). For BMI categories, these were defined as normal, overweight, obese, and underweight.

### Periodontal assessment

Periodontal examination was performed following the NHANES protocol and was defined according to (CDC/AAP). Severity of periodontal disease was determined by clinical measurements of probing depth (PD) and clinical attachment loss (AL) for six sites (disto-buccal, mid-buccal, mesio-buccal, disto-lingual, mid-lingual, and mesio-lingual buccal), excluding the third molars. Measurements were taken with a periodontal probe severity. Periodontitis severity was defined as severe (≥2 interproximal sites with CAL ≥6 mm and ≥1 interproximal site with PD ≥5 mm), moderate (≥2 interproximal sites with CAL ≥4 mm or ≥ 2 interproximal sites with PD ≥5 mm), and mild (≥2 interproximal sites with CAL ≥3 mm and ≥ 2 interproximal sites with PD ≥4 mm or ≥1 site with PD ≥5 mm). Periodontal status was categorized without periodontitis or with periodontitis, while periodontal severity was categorized as none, mild and moderate/severe (periodontitis). Bleeding on probing (BOP) was also calculated. About 20 s after probing, BOP was confirmed if the bleeding was detected at the lingual and/or buccal surfaces, respectively. BOP was classified as high for each individual if 30% or more of buccal and/or lingual surfaces showed BOP as previously described [14]. For the categories used in the oral mycological assessment for periodontal severity, we used for a range of no disease if BOP (0–9%), if BOP was between 10% and 29% it was considered mild and if >30% moderate to severe.

### Measures of environmental spore levels

Fungal spore data was obtained from the American Academy of Allergy Asthma and Immunology (AAAAI) San Juan station located in the Department of Microbiology of the Medical Sciences Campus of the University of Puerto Rico. For the enumeration of the outdoor spores, we used the 12-transverse-traverse methodology proposed by the British Aerobiology Federation [15]. Airborne spores were collected using a volumetric Hirst-type sampler, specifically a Burkard (Burkard Scientific Ltd, Uxbridge, UK). This equipment was located on the rooftop of the Medical Sciences Campus of the University of Puerto Rico, 30 m above ground level (Coordinates 18°23’53.7’ N, 66°04’25.3’ W). The Burkard 24-h trapping system worked continuously with an intake volume of 10 l of air/min. Fungal spores were impacted on a microscopic slide coated with a thin layer of 2% silicon grease as a trapping surface. The slide was changed daily and mounted on polyvinyl alcohol (PVA) mounting media for microscopic examination. Counting was done on each preparation along transverse fields every 2 h for 24 h on the longitudinal traverse. Spores were identified based on their morphological differences [16]. The identification was performed utilizing a bright-field optical microscope NIKON Eclipse 80i microscope (Nikon Manufacturing), using a magnification of 1000X.

Distribution of spore counts was done by executing the quartile function in Excel with defined spore abundances to scores, varying from 1 to 4. According to these obtained values, we distributed the outside spore levels range as Level 1 (6771–39243 m3), Level 2 (39489–49461 m3), Level 3 (50015–76854 m3), and Level 4 (77685–102986 m3). Spore count scores were added as metadata categories to the microbiota analyses. As the oral cavity connects with the nose and the esophagus and also with the breathing passages (trachea and lungs) spores can be detected in the oral cavity and allows one to detect both resident fungi and environmental spores.

### Genomic DNA extractions and ITS2 gene amplification and sequencing for oral mycobiome characterization

The University of Puerto Rico Biosafety (IBC, protocol # 49218) approved the applied laboratory protocols. Saliva (1.0 ml) was collected using sterile suction tubes and centrifuged at 13.2 rpm for 5 min, discarding the supernatant while the pellet was kept for the DNA extraction using standard protocols of the PowerSoil Kit (QIAGEN LLC, Germantown Road, Maryland, USA). DNA was quantified using the Qubit® dsDNA HS (High Sensitivity) Assay (ranging from 5 to 100ng/ul) at room temperature (Waltham, Massachusetts, US) and stored at −20°C, until further use.

The Internal Transcribed Spacer (ITS) is a region of nonfunctional RNA located between structural ribosomal RNAs 5.8S and 28S and is universally used to study fungal phylogeny [17]. The ITS2 genetic variability is useful in rapidly and accurately detecting fungal isolates. We used primers ITS9-FW (5′-GAACGCAGCRAAIIGYGA-3′) and ITS4-RV (5′-TCCTCC GCTTATTGATATGC-3′) for amplification and sequencing using Illumina Miseq, according to the Earth Microbiome Project protocols [18].

### ITS2 sequence analyses

Sequences obtained from the amplified ITS2 region were deposited and processed in QIITA [19] (project ID 13 using a Phred score above 30 (for quality control), deblurred and clustered into operational taxonomic units (OTUs) with 97% identity threshold. Taxonomy was assigned using UNITE ver 8.97 database [20], and sequences with more than 1,000 reads were included for analyses. Filtering of singletons (>3) was done in QIIME2 [21]. Additional analyses were done with two additional tables: we created a species table that included only environmental fungi, with a total of 43 samples after rarefaction (Supplementary Table 1.B), and another species table of fungi/yeast related to the oral cavity (Supplementary Table 2.B), with a total of 48 samples, after rarefaction.

Beta diversity distances and fungal community composition were analyzed by calculating pairwise Bray-Curtis distances between samples using *phyloseq* R package[27]. Dissimilarities among samples were visualized with a Principal Coordinate Analysis (PCoA) and Permanova [22], and Permdisp [23] measures were obtained using the qiime *beta_group_significance* command from QIIME2 [21].

Boxplots of alpha diversity measures, including richness (Chao1 Index) and evenness (Shannon Index), were created using R’s *phyloseq* [24] and *ggplot2* [25] packages. Chao1 and Shannon Index pairwise measures, implementing Kruskal–Wallis statistical tests, were obtained using the *ggpubr* package to compare all analyzed categories.

The QIIME [21, 26] platform was employed to obtain genus and species-level taxonomic profiles using the mean values. Significant taxa (p-value <0.05) were selected using the *group_significance.py* script in QIIME [21], which identifies significantly different OTUs using the Kruskal–Wallis statistical test. Boxplots were generated using the *ggplot2* [25] package in R [24]. Using QIIME [21] core microbiome script *compute_core_microbiome*, a core microbiome of 52% was calculated for healthy participants (without periodontal disease), while a core microbiome of 43% was obtained for participants with periodontitis. Bar plots depicting significant taxa were visualized through R package *ggplot2* [22].

Data availability statement: ITS-2 Sequence data supporting the analyses presented in the paper can be found in QIITA [19], project ID 13193, and publicly available with EBI accession number ERP126217 (https://www.ebi.ac.uk/ena/browser/view/PRJEB42371).

## Results

### Clinical characteristics

A total of 88 participants were recruited to this study. Most participants (94%, 83 out of 88) reported oral sex practices; however, 70% of the participants did not have periodontal disease, 10% had mild periodontitis, and 20% had moderate/severe periodontitis (Table 1). Detailed characteristics of periodontitis severity are presented in Table 1. Most participants with moderate/severe periodontitis were males (65%), and participants who had periodontal disease were aged 31–49 years (65%) and had good oral hygiene (42%).

**Table 1.** Clinical and behavioral characteristics by periodontal disease severity. Rarefaction level remains the same, samples with 1000 or more sequences were kept.

		No Severity = 62 (70%)					Mild Severity = 9 (10%)					Moderate/severe = 17 (20%)				
			Raw		Filtered			Raw		Filtered			Raw		Filtered	
Categories		N	Sequences	OTUs	Sequences	OTUs	N	Sequences	OTUs	Sequences	OTUs	N	Sequences	OTUs	Sequences	OTUs
Sex	female	30	469583	2220	469191	1828	3	28557	221	28517	181	6	151637	376	151575	314
	male	32	1010043	2357	1009596	1910	6	353641	512	353567	438	11	343282	1099	343078	895
Age	21–30	31	466144	2028	465781	1665	7	376158	599	376060	501	2	59394	98	59387	91
	31–40	25	540925	1988	540549	1612	2	6040	134	6024	118	6	225595	772	225452	629
	41–49	6	472557	561	472457	461						9	209930	605	209814	489
Oral hygiene	excellent	9	151670	690	151579	599	3	7591	215	7564	188	2	60114	232	60067	185
	good	36	956160	2538	955674	2052	3	206731	275	206685	229	8	275935	707	275803	575
	poor	17	371796	1349	371534	1087	3	167876	243	167835	202	7	158870	536	158783	449
Smoking	no	47	878041	3327	877458	2744	6	176195	456	176129	390	9	102631	591	102546	506
	yes	15	601585	1250	601329	994	3	206003	277	205955	229	8	392288	884	392107	703
Alcohol Consumption	no	9	30771	574	30701	504	2	143893	170	143862	139	6	45454	394	45364	304
	yes	53	1448855	4003	1448086	3234	7	238305	563	238222	480	11	449465	1081	449289	905
Marihuana Usage	no	32	717468	2548	717047	2127	6	359609	541	359523	455	9	116442	695	116334	587
	yes	30	762158	2029	761740	1611	3	22589	192	22561	164	8	378477	780	378319	622
												2	18824	148	18814	138
Oral Sex Practices	none	1	4588	82	4569	63	1	2287	74	2272	59	1	1662	50	1659	47
	yes	61	1475038	4495	1474218	3675	8	379911	659	379812	560	14	474433	1277	474180	1024
BMI	Normal	23	249728	1595	249438	1305	4	208282	356	208225	299	4	256550	301	256501	252
	Obese	19	442898	1398	442612	1112	1	17754	67	17748	61	8	45974	504	45911	441
	Overweight	17	779866	1358	779634	1126	2	6040	134	6024	118	4	181286	558	181161	433
	Underweight	3	7134	226	7103	195	2	150122	176	150087	141	1	11109	112	11080	83
***Total:***		***62***	***1479626***	***4577***	***1478787***	***3738***	***9***	***382198***	***733***	***382084***	***619***	***17***	***494919***	***1475***	***494653***	***1209***

A higher percentage of smokers had moderate/severe periodontitis (47%). Most participants with periodontal disease consumed alcohol. However, most participants with periodontal disease did not consume marihuana (Table 1).

From a total of 2,356,743 raw reads, 2,355,524 were used for analyses after removing singletons (>3) and unidentified taxa. From these reads, we obtained 5566 OTUs from 88 samples (Table 1). After rarefying our species table, all analyses were performed using the same number of reads per sample (1007 reads) for normalization purposes. Tables 2 and 3 depict the species table of environmental fungi and Candida species.

**Table 2.** Environmental species table statistic numbers of sequences and OTUs according to metadata categories. Rarefaction level remains the same, samples with 1000 or more sequences were kept.

Categories		N	%	Sum of Sequences	Sums of OTUS
Periodontal Severity	no	31	72.1	105560	991
	mild	4	9.3	6535	153
	moderate/severe	8	18.6	38993	287
Periodontal Status	without periodontitis	31	72.1	105560	991
	periodontitis	12	27.9	45528	440
Sex	female	19	44.2	43549	634
	male	24	55.8	107539	797
Smoking	non-smoker	29	67.4	106745	852
	smoker	14	32.6	44343	579
Alcohol Consumption	consumes alcohol	34	79.1	115963	1094
	doesn’t consume alcohol	9	20.9	35125	337
Marihuana Usage	doesn’t use marihuana	26	60.5	71846	887
	uses marihuana	17	39.5	79242	544
Outside Spore Levels	Spore Level 1	10	23.3	39625	308
	Spore Level 2	8	18.6	24726	287
	Spore Level 3	13	30.2	65009	483
	Spore Level 4	12	27.9	21728	353
Total		43	100	151088	1431

**Table 3.** Candida species table statistic numbers of sequences and OTUs according to metadata categories. Rarefaction level remains above 1000, samples with 1049 sequences or more were kept.

Categories		N	%	Sum of Sequences	Sum of OTUS
Sex	female	20	42	564469	351
	male	28	58	1542831	661
Age	21–30	22	46	792933	428
	31–40	16	33	681384	331
	41–49	10	21	632983	253
Periodontal Severity	no	34	71	1308718	690
	mild	4	8	363671	111
	moderate/severe	10	21	434911	211
Outside Spore Levels	Spore Level 1	10	21	223752	209
	Spore Level 2	8	17	92133	96
	Spore Level 3	15	31	887240	344
	Spore Level 4	15	31	904175	363
*Total*		*48*	100	*2107300*	*1012*

### Fungal communities according to periodontal severity

**Figure 1.**
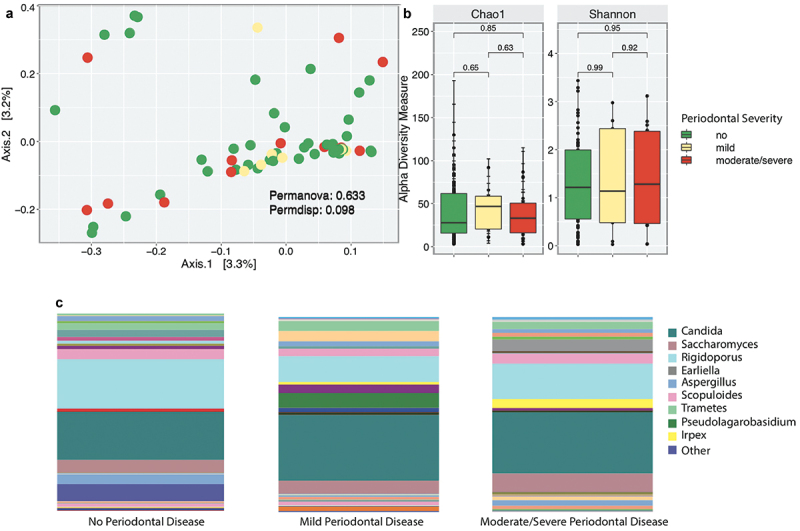
Fungal community profiles including beta (a), alpha diversity (b), and taxonomic profiles at genus level (c), according to the periodontal severity levels.

Mycobiota diversity was analyzed between participants without periodontitis (healthy), participants with mild and moderate/severe periodontitis (Figure 1). We also evaluated periodontal status (healthy participants vs. some level of periodontal disease), as supplementary analyses (Supplementary Figure S1). There were no significant differences in community structure, composition, or distance between samples among periodontal categories (Figure 1(a)). Supplementary analyses on periodontal status showed no significant differences in beta diversity (Supplementary Figure S1a).

Alpha diversity was estimated using Chao1 and Shannon Indexes, yet no significant differences were observed in richness or evenness of fungi present in the oral cavity (Figure 1(b)). Although not significant, we observed that participants with mild periodontitis were richer, when compared with healthy and moderate/severe participants (Figure 1(b)). Likewise, periodontal status showed no significant differences in richness nor diversity of species between participants with and without periodontal disease (Suplementary Figure S1b).

Taxonomic profiles were analyzed at the genus level (Figure 1(c)). The most abundant genera across all categories included *Candida, Saccharomyces, Rigidoporus, Aspergillus, and Trametes*. *Aspergillus*, an environmental fungus, was dominant in healthy participants, *Pseudolagarobasidium* was more abundant in participants with mild periodontal disease, while *Irpex* and *Saccharomyces* were more abundant in moderate/severe periodontitis.

**Figure 2.**
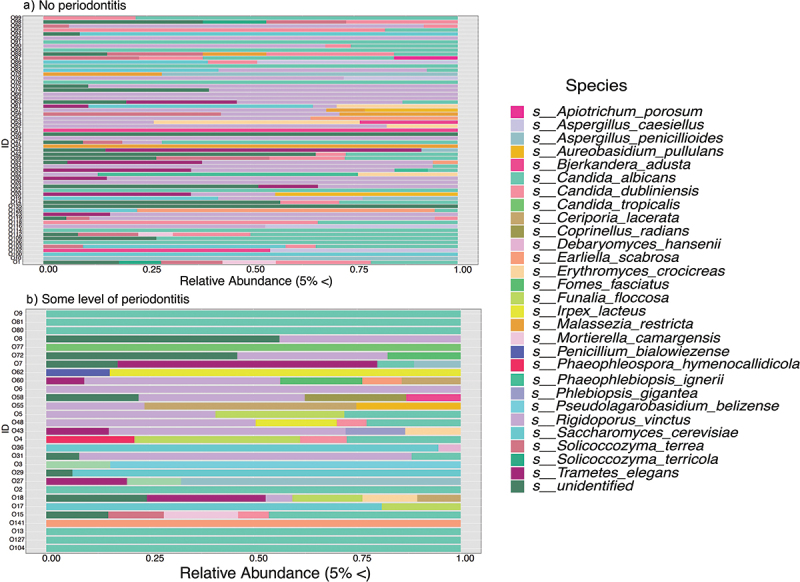
Comparison of the fungal core microbiome at species level, between healthy and diseased participants (some level of periodontis mild or severe).

A core microbiome at the species level was calculated for healthy and diseased participants. The healthy core microbiome that 43% of samples shared was mostly dominated by *Rigidoporus vinctus* and *Candida albicans* (Figure 2(a)). Species like *Candida dubliniensis, Saccharomyces cerevisiae, and Trametes elegans* were also observed at lower levels. On the other hand, the core microbiome calculated for OTUs shared by 52% of participants with some level of periodontal disease had a higher relative abundance of *Saccharomyces cerevisiae, Rigidoporus vinctus, and Irpex lactus* (Figure 2(b)*). Candida albican*s and many environmental fungi were observed across all periodontal severity categories.

**Figure 3.**
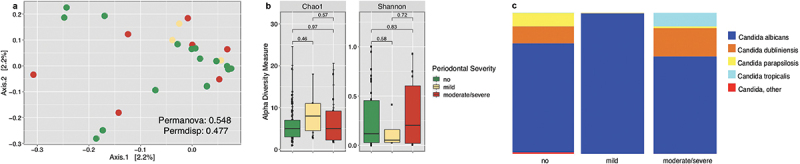
Diversity of *Candida* popularions. Beta diversity (a), alpha diversity (b), and taxonomic profiles (c), showcase the relative abundance of *Candida* species according to periodontal severity.

We evaluated the diversity and relative abundance of *Candida* species across all periodontal categories (Figure 3). No significant differences were found between *Candida* populations across the gradient of periodontal severity. Diversity analyses regarding community structure and composition (Figure 3(a)) revealed no significant differences (p-value >0.05). Alpha diversity was also not significantly different across periodontal severity (Figure 3(b)). *Candida albicans* was found to be ubiquitous. *Candida parapsilosis* was more abundant in healthy participants, while *Candida tropicalis* dominated in participants with moderate/severe periodontitis (Figure 3(c)). Although *Candida tropicalis* seems dominant in moderate/severe participants, only 2 participants had *Candida tropicalis* and dominated in sample ID O77. This participant is a male between 31 and 40 years of age, is overweight, and consumes alcohol, marihuana, and smokes.

### Changes to the mycobiota associated with alcohol consumption, marihuana usage, and smoking habits

**Figure 4.**
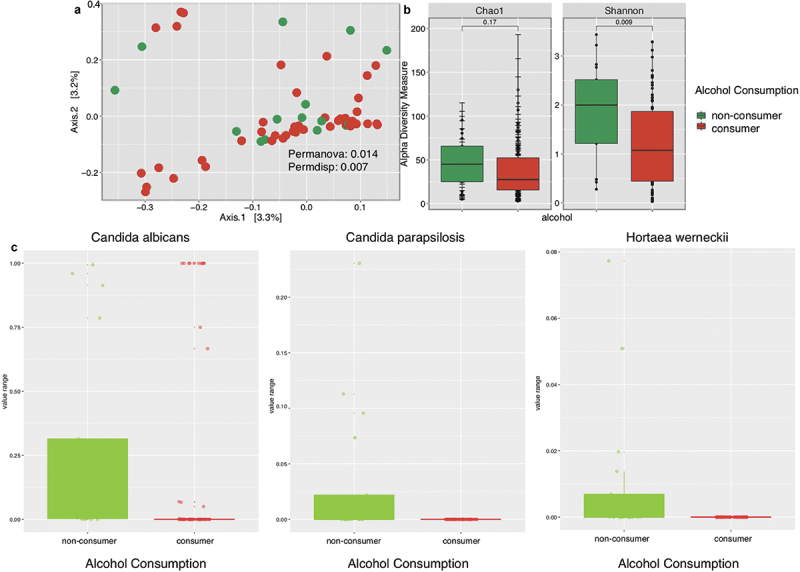
Fungal community profiles, including beta and alpha diversity, along with significant taxa at species level, according to alcohol consumption.

Alcohol consumption, marihuana usage, and smoking habits were considered the most influential risk factors impacting our cohort’s oral mycobiota. We discovered significant differences (Permanova: 0.014, Permdisp: 0.007) in fungal community composition and dispersion of samples between participants who consume alcohol and those who do not consume alcohol (Figure 4(a)).

Significant differences in alpha diversity were observed when assessing alcohol consumption. Non-consumers had higher overall species diversity (Shannon Index: 0.009) than consumers (Figure 4(b)). Boxplots of significant taxa (p-value <0.05) revealed *Candida albicans, Candida parapsilosis, and Hortaea werneckii* to be more distinctive in non-consumers (Figure 4(c)).

**Figure 5.**
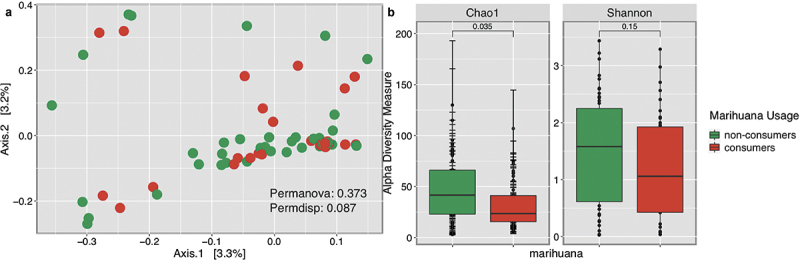
Diversity analyses comparing marihuana usage on participants, regardless of periodontal disease.

Changes in the mycobiota diversity based on marihuana usage showed no significant differences (p-values >0.05) in the community composition and structure of fungi present in the oral cavity (Figure 5(a)). Although differences in richness and overall diversity were not significant (p-values >0.05), non-users had higher Chao1 and Shannon Indexes (Figure 5(b)), demonstrating that they are richer and more diverse than those participants who use marihuana. No distinctive taxonomic profiles were found regarding marihuana usage.

**Figure 6.**
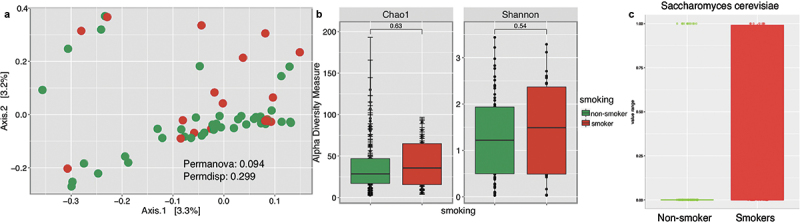
Fungal community profiles, including beta diversity plot (a), alpha diversity (b), and significant taxa at species level (c), between smokers and non-smokers.

Beta diversity of smokers, compared to non-smokers, showed no significant differences (p-values >0.05) in community composition and dispersion between samples (Figure 6(a)). Diversity analyses on richness and species diversity revealed no significant differences (p-values >0.05) between smokers and non-smokers (Figure 6(b)). However, when evaluating significant fungi (p-value <0.05), we found dominance of *Saccharomyces cerevisiae* in smokers (Figure 6(c)).

### Impact of environmental outdoor spore levels in fungi present in the oral cavity

Environmental outdoor spore levels representing atmospheric spore counts were measured for the days participants were recruited. Measured spore levels were divided into four categories, ranging from a low spore count in the outside environment (spore level 1) to a very high atmospheric spore count (spore level 4). These spore levels were associated with fungi in the oral cavity for a subset of participants, n = 43 for environmental fungi analyses (Table 2), and n = 48 for *Candida* species analyses (Table 3).

**Figure 7.**
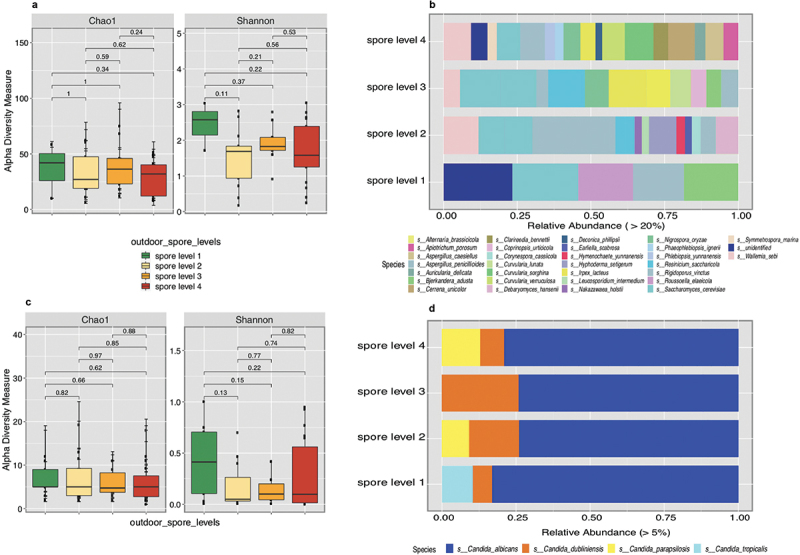
Plots depicting richness and evenness (a, c), along with relative abundance of environmental fungi (b) and *Candida* species in relation to outside spore levels (c).

Alpha diversity analyses of outdoor environmental fungi on the fungal communities of the oral cavity revealed no significant differences as outdoor spore levels increased. However, as atmospheric spore counts increased, these fungal communities’ richness and overall diversity decreased (Figure 7a). Oral taxonomic profiles of environmental fungi showed a higher abundance of *Cerrena unicolor*, *Curvularia, Apiotrichum*, and *Irpex lactus* with increasing outside spore levels (Figure 7b).

As outdoor spore levels increased, alpha diversity analyses of Candida species diversity showed no significant differences. However, there was a tendency for richness and decreased overall diversity as spore levels increased (Figure 7(c)). Analysis regarding oral taxonomic profiles revealed changes in the abundance of *Candida* species as outside spore levels increased (Figure 7(d)).

## Discussion and conclusions

Our data confirms the colonization of both yeast and filamentous fungi in the oral biofilm. Although we found no significant differences in structure, composition, or diversity between fungi in the oral cavity associated with disease progression, there were changes in composition among fungal species. The most abundant genera of fungi in the oral cavity included *Candida, Saccharomyces, Rigidoporus, Aspergillus, and Trametes*. Core mycobiome analyses found a higher abundance of *Rigidoporus vinctus* and *Candida albicans* in participants without periodontal disease. In contrast, *Saccharomyces cerevisiae, Rigidoporus vinctus, and Irpex lactus* were dominant in participants with periodontal disease. Interestingly, the presence of environmental fungi in the oral cavity for this population was mainly composed of basidiomycetes, representing over 60% of the outdoor fungal spores in Puerto Rico [28].

The ubiquity and abundance of *Candida* are of utmost importance for understanding oral health, as several *Candida* species cause oral candidiasis [29]. These yeasts are commonly found in the oral cavity, but the imbalance of this flora can lead to the development of candidiasis. *Candida* adheres to the epithelial cell membrane as its first step for infection, and it is aided by C3d receptors (a protein complex and T-cell receptor), mannoproteins, and mannose present in the cell wall [30]. Other virulence factors of *Candida* include endotoxins, proteinases, and inducement of Tumor Necrosis Factor (TMF) [30]. Several *Candida* species have been isolated from the oral cavity, Candida albicans being the most dominant. While *Candida albicans* is considered a commensal yeast in the oral cavity of healthy individuals, it can become pathogenic in immunocompromised individuals. It is mainly observed as opportunistic infections in individuals with diabetes, oral cancer, HIV, and periodontal disease [31]. Antibiotic use is also a risk factor for oral candidiasis, especially in people with HIV [32]. Aside from *Candida albicans, Candida dubliniensis, Saccharomyces cerevisiae*, and *Trametes elegans* were also detected. Like C. *albicans, C. dubliniensis* is a commensal yeast in the oral cavity associated with oral candidiasis [33]. *Saccharomyces cerevisiae* is considered a commensal fungus, mainly used in biotechnology [34,35]. Participants with periodontal disease presented a higher abundance of *Rigidoporus vinctus, Aspergillus penicillioides*, and *Saccharomyces cerevisiae. Aspergillus* species have been related to Aspergillosis, a fungal infection that can affect immunocompromised individuals and those with hematological malignancies [36]. *Aspergillus penicilloides* is a widespread xerophilic indoor fungus in Puerto Rico with allergenic potential [37]. *Candida albicans* and *Rigidoporus vinctus* – an environmental fungus – were found across all periodontal categories. An increase of outdoor environmental fungi in the oral cavity of participants with periodontal disease severity suggest the impact of the outside environment on the human microbiome and the impact of non-indigenous taxa on immune response and disease persistence.

When evaluating risk factors for periodontal disease, alcohol consumption showed a higher impact on fungal diversity. Non-consumers were richer and more diverse than those who consumed alcohol. Heavy alcohol consumption has been proposed as a risk factor for carcinogenesis due to the production of acetaldehyde, the first metabolite of ethanol [38]. It is believed that acetaldehyde in the oral cavity reacts with DNA, resulting in mutations that could lead to cancer development [39]. Also, studies have reported that individuals who consume high alcohol volume have higher levels of pro-inflammatory cytokines while also impairing macrophage function [36]. Evaluation of taxonomic profiles showed that alcohol consumers had dramatic decreases in *Candida albicans*, *Candida parapsilosis*, and *Hortaea werneckii* as compared to non-alcohol consumers. However, further studies need to identify possible biomarkers associated with healthy individuals.

We noticed how the use of marihuana decreased the richness and diversity of fungal communities present in the oral cavity. This finding implies the possible effect of marihuana on fungal community biofilm and might suggest that medicinal cannabis could positively impact oral fungal infections such as candidiasis. Nonetheless, this inhibition is not specific, and other studies have indicated mycosis due to marihuana usage [40]. While no significant differences were observed in diversity nor community composition by alcohol consumption, *Saccharomyces cerevisiae* was more abundant in smokers than in non-smokers [41]. Even though *Saccharomyces cerevisiae* is considered a ubiquitous yeast and used as a probiotic [35], infections have been reported in immunocompromised individuals, ranging from fungemia to skin infections and esophagitis [42,43].

Due to the high abundance of environmental fungi in the oral cavity, we then identified the effect of outdoor spore levels with fungi present in the oral cavity and spore counts corresponding to the participant recruitment dates. This analysis revealed a decrease in both the richness and overall diversity of fungi in the oral cavity as outside spore levels increased. We believe that this may be due to competition between environmental spores with mucosal yeast and the oral cavity filamentous fungi, ultimately reducing its abundance. For instance, *Candida* must adhere to epithelial surfaces to stay in the oral cavity [44,45], and environmental spores entering the oral cavity as we breathe and speak, may interfere with this adhesion mechanism. Additionally, we may speculate that inhaled outdoor fungi may induce an inflammatory response inhibiting the oral mucosa biofilm.

The ability of fungi to successfully adhere to the oral cavity, including the fungi acquired from the environment leading to the formation of hyphae, could increase surface hydrophobicity, the secretion of hydrolytic enzymes and toxins, thus enabling the attack on host cells, likely impact bacterial composition, by increasing inflammation and leading to oral dysbiosis.

Our data shows that associated risk factors such as alcohol consumption, marihuana usage, smoking, and the outdoor environment impact the oral mycobiome however these are not generalizable to population level. Some limitations included the limited age-range of 21–49 years old, the recruitment setting of STI clinics and therefore limited representation of healthy young adults. Even though this study has reduced confounding variables and batch effects which could mask biological signals, we recognize that a lack of mycobiome-associated signal associated with periodontitis could be caused by low power rather than a lack of real biological signal. Other pilot studies on oral mycobiome and on bacterial communities included the recruitment of low sample sizes with no clear exclusion criteria [46,47], a difficulty inherent to many microbiome studies, especially in low resource settings.Although not generalizable, still, it adds to our knowledge of oral microbiome dynamics. The role of fungal co-existence with bacterial periodontopathogens has been demonstrated before with *Candida albicans* shown to cohabit with *Porphyromonas gingivalis*, and other strictly anaerobic bacteria [48]. No other study has co-investigated oral and environmental microbes and shed light on the possible ecological competition of environmental spores with local oral epithelial microbiomes. Our data shows that associated risk factors such as alcohol consumption, marihuana usage, smoking, and the outdoor environment impact the oral mycobiome.

## Supplementary Material

Supplemental Material

Suppl_fig_1.jpg
