# Supplementary material for: Ecological competition in the oral mycobiome of Hispanic adults living in Puerto Rico associates with periodontitis
Source: J Oral Microbiol. 2024 Feb 21;16(1):2316485. doi: 10.1080/20002297.2024.2316485 (PMC10883086; doi:10.1080/20002297.2024.2316485)
Supplement: Supplemental Material [file ZJOM_A_2316485_SM0579.docx]

S**upplementary figure 1:** Diversity analyses comparing healthy participants (no periodontal disease), versus participants with some level of periodontal disease
