# Supplementary figures and images for: Ecological competition in the oral mycobiome of Hispanic adults living in Puerto Rico associates with periodontitis
Source: J Oral Microbiol. 2024 Feb 21;16(1):2316485. doi: 10.1080/20002297.2024.2316485 (PMC10883086; doi:10.1080/20002297.2024.2316485)

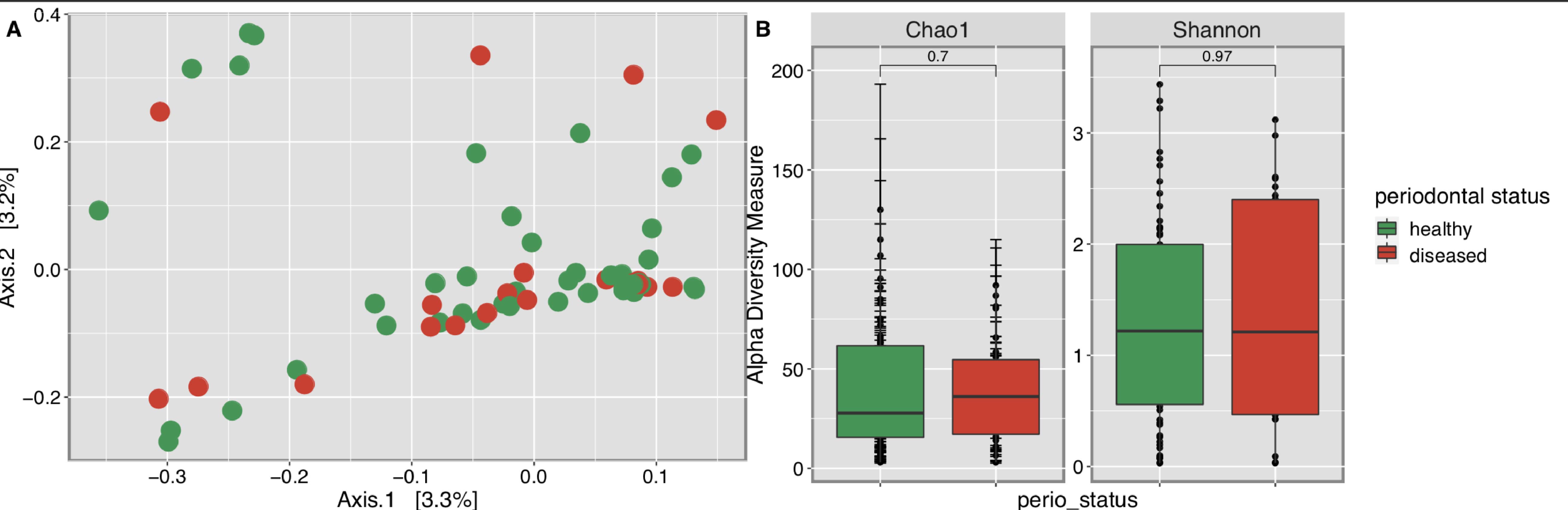

Supplement: Suppl_fig_1.jpg [file ZJOM_A_2316485_SM4791.jpg]
